# COVID-19 and the Elderly’s Mental Illness: The Role of Risk Perception, Social Isolation, Loneliness and Ageism

**DOI:** 10.3390/ijerph19084513

**Published:** 2022-04-08

**Authors:** Anna Rosa Donizzetti, Martine Lagacé

**Affiliations:** 1 Department of Humanities, University of Naples Federico II, 80133 Naples, Italy; 2 Department of Communication, University of Ottawa, Ottawa, ON K1N 6N5, Canada; martine.lagace@uottawa.ca

**Keywords:** mental illness, ageism, COVID-19 age discrimination, loneliness, social isolation, COVID-19 risk perception, fear of COVID-19, COVID-19 outbreak

## Abstract

For almost two years, populations around the globe faced precariousness and uncertainty as a result of the COVID-19 pandemic. Older adults were highly affected by the virus, and the policies meant to protect them have often resulted in ageist stereotypes and discrimination. For example, the public discourse around older adults had a paternalistic tone framing all older adults as “vulnerable”. This study aimed to measure the extent to which perceived age discrimination in the context of the COVID-19 pandemic, as well as the sense of loneliness and social isolation, fear and perception of COVID-19 risks, had a negative effect on older adults’ mental illness. To do so, a self-report questionnaire was administered to 1301 participants (average age: 77.25 years old, SD = 5.46; 56.10% females, 43.90% males). Descriptive and correlational analyses were performed, along with structural equation modelling. Results showed that perceived age discrimination in the context of the COVID-19 pandemic positively predicts loneliness and also indirectly predicts mental illness. In addition, loneliness is the strongest predictor of mental illness together with fear of COVID-19 and social isolation. Such results highlight the importance of implementing public policies and discourses that are non-discriminating, and that favour the inclusion of older people.

## 1. Introduction

The “new coronavirus disease” better known as COVID-19 [1] originated in Wuhan, China at the end of 2019 and then expanded globally. On 11 March 2020, the World Health Organization declared it a pandemic status, a pandemic that is still ongoing. During these past two years, we have observed various phases of the pandemic with different degrees of propagation, virulence and lethality, due, on the one hand, to the multiplication and spread of different variants of the virus and, on the other hand, to the roll out of vaccines and the implementation of sanitary measures. The various measures to contain the spread of the virus, such as social isolation, the adoption of appropriate hygiene measures, the use of masks, physical distancing, temperature measurement, the suspension of activities in cultural centres, etc., were used at varying times and with varying rigor. From the outset, it was understood that to reduce the propagation of COVID-19, contact between people had to be reduced as much as possible, so much so that the Italian government, in the first phase of the virus’s spread, decided to adopt a strict containment measure, i.e., the closure of all non-essential or strategic production activities [2]. From 9 March to 4 May, only supermarkets, pharmacies, shops selling necessities and essential services remained open. Almost two months of complete physical isolation led to many psychological problems such as increased stress and depression [3].

The segment of the population most affected by the virus was the older individuals; because of the high morbidity and mortality rates in old age, the COVID-19 pandemic was considered a ‘geropandemic’ [4]. Concurrently, the older people were also those most affected by the restrictions. In fact, a message that has constantly been transmitted, in Italy and other countries, in the communiqués of heads of government and by the media in general, has been the recommendation to the elderly or those suffering from chronic or immunodepression pathologies to go out of the house only if necessary and to avoid frequenting crowded places [5]. Thus, the COVID-19 pandemic accentuated the social isolation of older individuals, resulting in their exclusion from social life [6]. While the restrictions were intended to be protective, such policies have often resulted in paternalistic public communication describing all older people as “vulnerable” members of society [7]. This has led to the re-emergence of already existing stereotypes of the elderly who were treated as one homogeneous segment of the population. Governments, health professionals, the media and social networks have portrayed the elderly as a burden on society; ageing has been associated with decline, worthlessness and taking charge. Such attitudes have reinforced intergenerational conflicts, prejudice and discrimination [8].

The general objective of the present study is to investigate the mental illness of older people during the COVID-19 pandemic. Mental illness refers not only to the presence or absence of disease but to all conditions that affect cognition, emotion and behaviour. Furthermore, we want to investigate the role that different factors may have on mental illness in older people, such as the perception of being discriminated against because of one’s age or/and the feeling of loneliness, social isolation, the fear of COVID-19 and the perception of risks related to COVID-19. It is very important to understand the role played by these factors to adopt sanitary measures, related messaging that will be transmitted in the later phases of this pandemic, as well as in future emergencies.

## 2. Theoretical Background

One of the most important goals facing society today is the ageing of the population, a phenomenon that is most evident in industrialised countries where it is most pronounced. The demographic revolution that has taken place in recent decades has generated a strong focus on the bio-psycho-social factors associated with the ageing processes.

Numerous studies have shown that social isolation and loneliness have strong negative consequences for physical and mental health [9,10,11,12]. Physical problems include cardiovascular diseases [13], lung diseases and arthritis [14], Alzheimer’s [15], as well as an association with mortality after many years [16,17]. Among the mental problems, there are higher levels of depression and psychological distress [18], despair [19] and thoughts of suicide [20]; more generally, it interferes negatively with quality of life of the older people [21]. In addition, an association with poorer cognitive functioning has also been established [22]. While social isolation and loneliness have often been used and reported in an undifferentiated way, they refer to different aspects [23] and, as numerous studies have shown, are also only weakly related [24,25]. Social isolation refers to the structure of the social network, which reflects the objective state of lack of social relationships [26,27]. Loneliness, however, is the feeling of lack or loss of a companion; it is, therefore, a subjective phenomenon that reflects the quality of a person’s social interactions [28]. Therefore, loneliness develops when one’s social relationships are not accompanied by the desired degree of intimacy [27,28]. According to the Discrepancy perspective of loneliness, loneliness occurs when there is a discrepancy between the quality and/or quantity of social relationships that people have [29]. Consequently, a person may feel lonely despite having a dense network of social relations, as well as, on the contrary, he or she may be socially isolated and not feel lonely [30]. At the same time, social isolation does not lead to loneliness when the desired level of social relationship is low [31]. Several risk factors are associated with loneliness, such as: Not being married/not having partners and loss of partner; a limited social network; a low level of participation in social activities; poor perceived health; and depression/depressed mood [32]. While little investigated, there is another important risk factor for loneliness in old age: Ageism [30].

The term ageism refers to stereotypes, prejudice and/or discrimination against people based on their age. Most studies have focused on the manifestations affecting older people, although it can manifest itself against younger people as well. Iversen, Larsen and Solem [33] defined ageism as “negative or positive stereotypes, prejudice and/or discrimination against (or to the advantage of) older people on the basis of their chronological age or on the basis of a perception of them as being ‘old’ or ‘elderly’. Ageism can be implicit or explicit and can be expressed on a micro, meso, or macro-level” (p. 15). Middle-aged adults possess higher levels of ageism [34] and have a greater sense that life is coming to an end, as well as higher levels of anxieties related to ageing, dying and death [35]. In Italy [36], males and young people have higher levels of ageism than women and older people. Moreover, recent studies have shown that the basis of negative stereotypes is mainly the poor knowledge of the ageing process. Lack of knowledge and a high level of anxiety about ageing are antecedents of stereotypes, which in turn, together with age, influence ageism [37].

Regarding the relationship between mental health and ageism, there are different studies available. A review [38] of the relationship between ageism and health revealed a significantly positive association between perceived ageism and mental health, physical/functional health and quality of life. Perceived age discrimination is generally described as one of the major experiences of discrimination in life [39]. This perception is subjective as people’s behaviour can be interpreted differently depending on the situation. An individual is more likely to perceive discrimination in a situation where they expect to be stereotyped negatively than in a situation where they expect to be stereotyped positively. Consequently, particularly negative age stereotypes provide a guideline for situations in which age-discriminatory behaviour is likely to occur [40]. The perceived age discrimination is also correlated with negative age stereotypes, as these can influence the behaviour of older people themselves, who perceive themselves to be the object of such stereotypes. According to Levy’s Stereotype Embodiment Theory [41], stereotypes present in culture are internalised, resulting in self-definitions which, in turn, influence functioning and health. The self-perceptions of ageing, that is, the endorsement of stereotypes about older people by people as they age, significantly predict negative effects on physical and mental functioning, sometimes even decades later [41]. Similarly, positive self-perceptions of ageing are correlated with better functional health more than two decades later [42]. This means that the stereotypes that people assimilate from the surrounding culture and identify with can act as self-fulfilling prophecies [17]. Furthermore, Sutin et al. [43], as part of the American Health and Retirement Survey, investigated the relationship between perceived discrimination and subsequent loneliness in a nationally representative sample of adults aged 50 years and over. The 7622 participants (mean age 67.5) took part in a longitudinal study and responses found that perceptions of age discrimination significantly predicted feelings of loneliness at five-year intervals. It has also been shown that stereotypes of loneliness in later life can become self-fulfilling prophecies, with the study by Pikhartova et al. [44] showing that both expectations and stereotypes of loneliness in old age predicted feelings of loneliness several years later.

In addition to ageism, loneliness and poor social relationships, other factors that may contribute negatively to the mental health of older adults include fear of contracting COVID-19 and perceived COVID-19 risk.

Fear of COVID-19 and feeling personally at risk of contracting the virus may also contribute to the mental stability of older people during this pandemic period. The available literature on past viral epidemics has already highlighted the role played by fear and its negative psychosocial consequences in exacerbating the damage of an infectious disease [45]. Indeed, fear can lead people to denial or phobia, as well as stigmatisation of citizens perceived as the source of the disease [45,46]. Moreover, an association between fear and other psychological disorders such as anxiety and depression has emerged, further affecting people’s quality of life in a negative way [47]. These consequences are even more relevant in the context of the current pandemic, where social isolation caused by government policies to contain the virus has already been the cause of a strong increase in symptoms of anxiety and depression, including amongst older populations [48,49]. Related to the fear of COVID-19 is the perception of COVID-19 risk [50]. Risk perception refers to people’s intuitive assessments of the dangers to which they are or might be exposed [51]. It involves subjective judgements that individuals develop based on the characteristics, severity and manner in which risk is managed and includes individuals’ psychological assessments of the likelihood and consequences of an adverse outcome [52]. Recent studies have shown that older people estimated the risk of COVID-19 to be less dangerous than younger people [53,54]. In addition, public understanding of risk could be a determinant of community mental and physical health [55,56].

### The Current Research

The current study aimed to investigate the relationships between mental illness, social isolation, loneliness, perceived age discrimination in the context of the COVID-19 pandemic, fear of COVID-19 and COVID-19 risk perception. Specifically, the following hypotheses were formulated:

**Hypothesis** **1.** *We expected that the restrictions imposed by the government resulted in an impoverishment of the network of meaningful social relationships (H1a); that the impoverishment of the network of meaningful social relationships was only partially mitigated by the possibility of using technology to contact family and friends (H1b); and that the social isolation index would be correlated with the loneliness measure precisely because of the impoverishment of social relationships (H1c).*

**Hypothesis** **2.** *We expected that mental illness was positively correlated with measures of perceived age discrimination in the context of the COVID-19 pandemic, loneliness, social isolation, COVID-19 risk perception and fear of COVID-19 (H2).*

**Figure 1:**
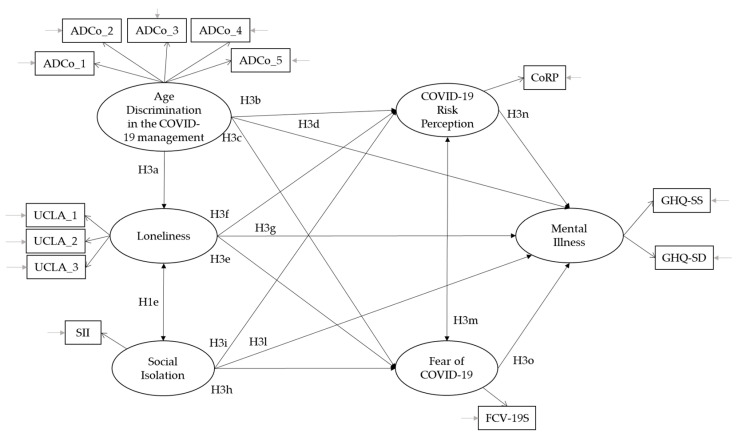
Conceptual Model.

**Hypothesis** **3.** *Based on the literature review, we constructed an a priori model to be tested. As shown in Figure 1, we expected that perceived age discrimination in the context of the COVID-19 pandemic would positively predict loneliness (H3a), COVID-19 risk perception (H3b), fear of COVID-19 (H3c) and mental illness (H3d); that the feeling of loneliness would positively predict fear of COVID-19 (H3e), COVID-19 risk perception (H3f) and mental illness (H3g); that social isolation would positively predict fear of COVID-19 (H3h), COVID-19 risk perception (H3i) and mental illness (H3l); that COVID-19 risk perception would be correlated with fear of COVID-19; and that COVID-19 risk perception and fear of COVID-19 would positively predict mental illness.*

**Hypothesis** **4.** *Finally, we expected that in addition to the direct effect of perceived age discrimination in the context of the COVID-19 pandemic on illness, there was also a mediating effect of loneliness (H4).*

## 3. Materials and Methods

### 3.1. Procedure of Recruitment and Participants

Participants were contacted through snowball sampling. A group of university students were asked to contact their older people acquaintances and through word of mouth other participants were reached. The only criterion for inclusion was being over 65 years old. Participants were asked to respond to a questionnaire about their perceptions during the pandemic from COVID-19. Data collection was initiated in the middle of May and ended at the start of June 2021. Participants completed a self-report survey that took approximately 30 min of their time. They were invited to fill out an online questionnaire connecting to a weblink associated to the Google Forms platform. Participation in the study was voluntary and anonymous and participants could drop out of the study at any time. The study protocol was approved by the Local Ethics Committee of the institution of the principal investigator, and the study was conducted according to APA ethical standards. The study also conformed to the ethical principles of the 1995 Helsinki Declaration. The first page of the survey asked for informed consent. The next page of the survey consisted of a presentation of different instruments: Loneliness Scale, General Health Questionnaire, Age discrimination in the COVID-19 management, Social Isolation Index, COVID-19 Risk Perception and Fear of COVID-19 (for a detailed description of the instruments, see the following section). The survey also included a short demographic section in order to collect information regarding the participant’s age, sex, marital status, religious faith and practice, educational level as well as a series of questions concerning the extent and frequency of the social relations network and participation in recreational activities.

The convenience sample consisted of 1301 Italian participants (56.1% females, 43.9% males), aged from 67 to 99 years old (M = 77.25, SD = 5.46); 57.10% were married or lived together, 38.20% were single and 2.20% were divorced or separated; 25.10% of participants lived alone, while the remaining 74.90% lived with at least one family member (M = 2.83; SD = 1.31).

Catholic participants made up 94.40% and stated that they practice their faith always (44.20%) or sometimes (44.00%); only 11.80% stated that they never practise their religious faith. The level of education was low, with 49.80% with a primary school qualification, 25.00% with a junior high school qualification and 18.70% with a high school qualification; a small percentage had an undergraduate (5.70%) or postgraduate (0.80%) degree.

### 3.2. Instruments

The various measures used in the survey are described below: 

**General Health Questionnaire_12** (GHQ) [57,58]. The General Health Questionnaire is aimed at detecting, even in the older population, common symptoms which are indicative of the various syndromes of mental illness (e.g., “Have you recently been thinking of yourself as a worthless person?”), differentiating individuals with psychopathology from those considered normal. The scale consists of 12 items with a four-point rating scale, ranging from 1 (not at all) to 4 (much more than usual). Subscales are somatic symptoms and social dysfunction, but for this study, only the overall measurement was considered. The internal reliability was 0.84 in this study.

**Age discrimination scale in the context of the COVID-19 pandemic** (ADCo). Perceived age discrimination about the management of COVID-19 was measured using five items assessed on a Likert scale from 1 (strongly disagree) to 5 (strongly agree). The items were adapted from Garstka’s scale [59] and are as follows: “I feel I am victim of the government’s Coronavirus policies because of my age”; “In this pandemic period, members of my age group have been discriminated against more than members of other age groups”; “During this pandemic period, members of my age group did not receive the same care as members of other age groups because of their age”; “In this pandemic period I feel that I have been deprived of the opportunities for others because of my age”; “I feel that social media (news, newspapers, etc.) has discriminated against me and members of my group because of the way the pandemic and its effects have been presented”. The mean of these five items was calculated, with higher values representing greater perceived discrimination. In the current study, the Cronbach’s α of the scale is 0.83.

**UCLA Loneliness Scale-version 3** (UCLA) [60,61]. The UCLA Loneliness Scale consists of 20 items for the global measurement of loneliness (e.g., “I feel isolated from others”). The items are evaluated on a four-point Likert scale, ranging from 1 (never) to 4 (always). Nine items are positively formulated and reversed. The scale consists of three dimensions (Isolation, Relational Connectedness and “Trait” Loneliness), but for this study, only the overall measure was considered. In this study, the internal consistency reliability of the scale is 0.88 (Cronbach’s α).

**Social Isolation Index (SII).** In line with the work of Wister et al. [62], to obtain a measure of social isolation, an index was calculated by averaging the following variables: Social network quantity, attendance in presence in the last month, attendance through technology in the last month and community participation (Table 1). In turn, these variables were calculated as follows: Social network quantity corresponds to the average of the answers given to items a, b, c, d, e, f and g (for frequencies see (a) in Table 1); the variable attendance in presence in the last month is the result of the average of the answers to items h, i, l, m, n and o (see (b) in Table 1); the variable attendance through technology in the last month derives from the average of items p, q, r, s, t and u (see (c) in Table 1); and the variable community participation was calculated by averaging items v, w, x, y and z (see (d) in Table 1). The scores were inverted so that high values correspond to high levels of social isolation.

***Fear of COVID-19 Scale*(FCV-19S)** [63]. The FCV-19S is a seven-item self-report questionnaire for which respondents must provide their degree of agreement on a five-point Likert-type scale (from 1 = “strongly disagree” to 5 = “strongly agree”). The scale, with a single-factor structure, is designed to assess the emotional, cognitive, physiological and behavioural manifestations of fear related to COVID-19 (e.g., “I am very afraid of coronavirus-19”, “When I look at news and stories about coronavirus-19 on social media, I get nervous or anxious”). Higher values indicate a greater fear of COVID-19. In this study, FCV-19S has a high internal consistency (in this study, Cronbach’s alpha, = 0.89).

***COVID-19 Risk Perception Scale*(CoRP)** [50]. The perception of COVID-19 risk was measured using four items (e.g., “Are you worried about getting diseased with COVID-19 yourself?”; “Are you worried about your family getting infected with COVID-19?”) assessed on a five-point Likert scale (from 1 = strongly worried to 5 = not worried at all). The items were then reversed so that high scores correspond to high levels of risk perception. In the current study, Cronbach’s alpha is 0.78.

### 3.3. Statistical Analysis

The survey data were entered into SPSS 22.0 databases and M-Plus 6.1 software. Cronbach’s alpha was used to calculate the reliability of the scales. For the psychological scale, an internal consistency should be greater than 0.70 (however, an alpha between 0.60 and 0.69 is considered acceptable [64]).

Descriptive statistics were first performed on data, including the Wilcoxon test for paired samples to assess differences between groups. Pearson’s correlation coefficients were also calculated to determine associations between variables under study. Second, Structural Equation Modeling (SEM) [65] was conducted to test the hypothesised model. To assess goodness-of-fit of that model, the following indicators were used: Chi-squared distribution and the degrees of freedom (χ^2^/df ≤ 3), although this index is sensitive to sample size; Standardised Root Mean Square Residual (SRMR ≤ 0.09); Comparative Fit Index (CFI > 0.90); and Tucker–Lewis index (TLI > 0.90). If the results of the Root Mean Square Error of Approximation (RMSEA) are ≤ 0.05 they are considered to be good, and reasonable if they are ≤ 0.08, with a cut-off value of 0.06. In order to assess the goodness of the model, several indices will be considered simultaneously as the different indices assess different aspects of the goodness-of-fit [66,67,68,69]. Satisfactory models should show consistently good-fitting results on many different indices.

## 4. Results

### 4.1. The Network of Social Relations

Participants were asked to indicate how often they saw members within their network of significant relationships before COVID-19 and during the last month, that is, during the COVID-19 outbreak period (Table 2).

Comparison of the averages using the Wilcoxon test showed that there was a significant reduction in attendance, especially of friends (before COVID = 2.40 vs. last month = 1.46), brothers/sisters (before COVID = 1.92 vs. last month = 1.21) and nieces and nephews (before COVID = 3.41 vs. last month = 2.77).

Interactions in presence during the last month and attendance through the use of technology were then compared and again significant differences emerged (Table 3).

In particular, frequentation through technological means exceeded ‘in presence’ only in the case of siblings (1.86 vs. 1.21) and friends (1.80 vs. 1.46) while attendance in presence always prevailed with neighbours (2.11 vs. 0.96).

### 4.2. Means, Standard Deviations and Correlation Analysis

Means, standard deviations and correlations are shown in Table 4.

### 4.3. Testing of the Hypothesised Conceptual Model

**Figure 2:**
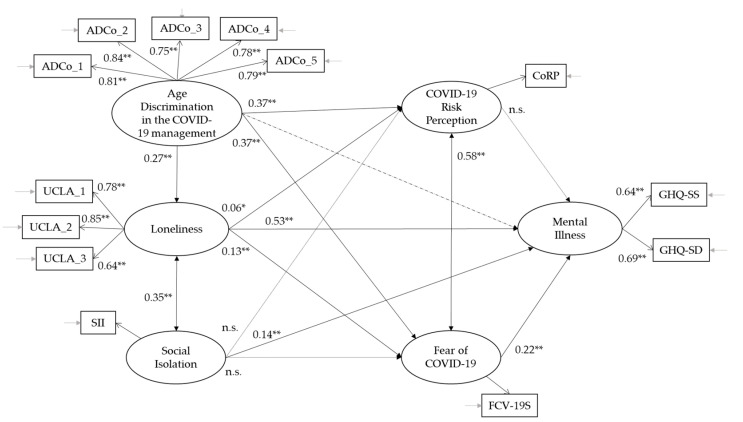
Structural equation models with standardised coefficient estimates (** *p* < 0.001; * *p* = 0.05; n.s. = not significant).

We used structural equation modeling to test the hypothesised relationships between variables under study (see Figure 2). Results suggest an acceptable fit between the theoretical and the empirical models: χ^2^(df) = 269.651 (53), *p* ≤ 0.001; χ^2^/df = 5.087; CFI = 0.969; TLI = 0.954; RMSEA = 0.056 (0.050–0.063); SRMR = 0.030. Not all the hypothesised relationships were significant. Precisely, as hypothesised, perceived age discrimination in the context of the COVID-19 pandemic positively predicts loneliness (H3a; β = 0.27 **), COVID-19 risk perception (H3b; β = 0.37 **) and fear of COVID-19 (H3c; β = 0.37 **). Loneliness positively predicts fear of COVID-19 (H3e; β = 0.13 **), COVID-19 risk perception (H3f; β = 0.06 *) and mental illness (H3g; β = 0.53 **). Loneliness also correlates with social isolation (H1e; β = 0.35 **), and social isolation predicts mental illness (H3h; β = 0.14 **). Fear of COVID-19 correlates with COVID-19 risk perception (H3m; β = 0.58 **) and predicts mental illness (H3o; β = 0.22 **).

In contrast to the hypothesis, social isolation does not predict COVID-19 risk perception and fear of COVID-19. Furthermore, perceived age discrimination in the context of the COVID-19 pandemic and COVID-19 risk perception do not predict mental illness.

While perceived age discrimination in the context of the COVID-19 pandemic does not directly affect mental illness (dashed lines in Figure 2), two indirect effects emerged. Specifically, perceived age discrimination in the context of the COVID-19 pandemic affects mental illness through the mediation of loneliness (H4; β = 0.14 **) and fear of COVID-19 (β = 0.08 **).

## 5. Discussion

The COVID-19 pandemic has been keeping humanity in a precarious and uncertain situation for close to two years. Based on the different phases that this pandemic has undergone and the scientific knowledge that has accumulated, governments have defined various measures that they consider to be the most appropriate to contain the spread of the virus. The persistence of the emergency has made it increasingly necessary to find a balance between containment measures and the need to continue the social and economic life of citizens. In this respect, the studies that are trying to capture the psychological effects of these measures are of fundamental importance so that this too is taken into account when defining the future actions of governments both in this pandemic and in any subsequent emergencies. The aim of this study was precisely to investigate the effects on the mental illness of older people of certain variables, such as social isolation, loneliness, perceived age discrimination in the context of the COVID-19 pandemic, fear of COVID-19 and risk perception of the COVID-19.

The hypotheses formulated have been partially confirmed. According to the first hypothesis, it emerged that the COVID-19 outbreak measure has generated an impoverishment of the network of significant social contacts for older people. Living in a technological era has only partially attenuated this condition of distancing from social life, accompanying a feeling of loneliness already particularly felt in this section of the population. In fact, in line with the existing literature [24,25], social isolation is moderately correlated with loneliness, although to a greater extent than in previous studies [70,71]. Concerning the second hypothesis, both the positive correlation between mental illness, loneliness and social isolation [11,72] and the positive correlation between mental illness, COVID-19 risk perception and fear of COVID-19 [73] were confirmed. In this regard, a particularly interesting finding is the positive correlation between mental illness and the measure of perceived age discrimination in the context of the COVID-19 pandemic, as well as between the latter and loneliness. The tested model has good indices of adaptation. The hypotheses we formulated regarding the relationships were partially confirmed. The model shows that the strongest antecedent of mental illness is loneliness, confirming a link that has already emerged in the literature [19]. Social isolation is also an antecedent of mental illness, but to a lesser extent than loneliness, confirming that these are two different but related constructs [26]. Most of the contacted older people do not live in social isolation as they mostly live with other family members, but at the same time, the impoverishment of extra-familiar relationships caused by the anti-COVID-19 norms may have contributed to exacerbating a feeling of loneliness which, already normally is strongly felt among older people. In addition, the feeling of loneliness and not the lack of social relationships puts older people in a more vulnerable position, with increased fear of contracting the virus and a higher perception of the risks associated with it. Analyses also show that feelings of loneliness are stronger in those who have perceived policies and discourses to protect the older population as discriminatory [30]. These perceptions did not directly affect people’s mental health but indirectly did, through the mediation of loneliness and fear of COVID-19. The perception of having received differential treatment due to one’s age also contributed to an increase in the perception of being at greater risk of contracting the virus, which then correlates with a greater fear of falling ill with COVID-19. Therefore, as in previous pandemics, fear hurt people’s mental health in the case of the coronavirus pandemic [45]. However, the same cannot be said of the perception of risk, which does not appear to be an antecedent of mental illness. This finding needs to be better investigated because, on the one hand, the perception of risk is correlated with fear and, on the other hand, it does not predict mental illness either negatively, as had been hypothesised, or positively in its traditional protective function for health with the concomitant implementation of self-protective behaviours.

One of the merits of this study is that it simultaneously considered several variables that had previously been considered separately, providing an overview of the relationships between the constructs investigated. Furthermore, it provided a clear picture of the factors that affected the mental health of older people during the COVID-19 pandemic, providing useful indications for policymakers. At the same time, there are limitations. Firstly, it is a cross-sectional study and this prevented the examination of causal relationships over time. Second, it is a single-method study, since only self-report instruments were used, and this may have led to the inflation of an observed association. Thirdly, the snowball sample, in addition to not being representative of the population, may have led to finding too homogeneous a group of subjects.

## 6. Conclusions

The COVID-19 pandemic confronted all humanity with an unknown evil, and all available resources were used to combat it. Sometimes it was necessary to take drastic decisions such as social isolation, which served to limit the spread of the virus and contain the number of deaths, especially among the most fragile people. At the same time, however, this measure also had negative repercussions on people’s mental health [3]. Indeed, older people with higher levels of mental illness were those who experienced higher levels of loneliness, fear of COVID-19 and social isolation. The invitation to reduce social contacts was perceived by the elderly as detrimental to their person, a real form of discrimination linked to the generalisation of the condition of frailty to all those over 65. While there is a link between the presence of chronic illnesses and age, being chronologically old is not the same as being vulnerable and in a precarious state of health simultaneously [74]. The other discrimination perceived by older individuals relates to not having had the same opportunities for care as younger people, especially when the rate of hospitalisation was particularly high, and the health system was not able to meet all the demands for care from the population. In addition to this, we need to consider the role of social media (news, newspapers, etc.) in the presenting the pandemic and its effects. The media played a central role in propagating age stereotypes and negative attitudes towards older people [75] and older people’s perception of stereotypes and prejudices significantly predicted their feelings of loneliness [43]. An important contribution to the mental illness of older people was also made by the feeling of fear generated by an unknown disease that has claimed so many lives, especially among older people. Doctors, politicians and the media have continually emphasised the risk of COVID-19 for older individuals in particular, sowing an almost paralysing fear. The perception of being discriminated against because of one’s age affects COVID-19 risk perception, which normally plays a central role in motivating health protection behaviour in general [76,77] and during pandemics [78]. At the same time, however, it can also feed the feeling of fear through a process of reciprocal influence, which affects mental illness.

Therefore, it is clear that, while the COVID-19 outbreak strategy served to reduce the spread of the virus by protecting the physical health of older people, it also greatly undermined their mental health. The feeling of loneliness caused partly by social isolation and partly by the perception of being discriminated against in the management of COVID-19 because of their age is the main contributor to their mental health, along with fear. Future policies should take this into account and provide for interventions aimed at preventing loneliness both in normal and emergency conditions, including interventions aimed at developing social skills, enhancing social support, expanding opportunities for social interaction and recognising maladaptive social cognition [79]. A strategy can also be to promote inclusivity in social media and to make older people’s voices heard [80]. The participation of older people in social media, on the one hand, can increase their involvement in social interactions and, on the other hand, can be a way to recognise and give voice to the diversity that exists in this age group. Finally, reliable and relevant information about older people should be conveyed and stigmatisation and labelling of older people should be avoided [81]. Combating ageism requires a concerted effort by all stakeholders to convey positive messages associated with ageing and to create an environment of respect, empathy and solidarity toward older people, especially during the COVID-19 pandemic [80].

## Figures and Tables

**Table 1:** Descriptive statistics and mapping scores for social isolation index variables.

(a) Social Network Quantity	Value	Frequency	Mean (SD)	
Number of family members you live with	0 = 5+ family members 1 = 4 family members 2 = 3 family members 3 = 2 family members 4 = 1 family members 5 = 0 family members	5.10% 4.80% 6.90% 14.40% 45.30% 23.40%	3.60 (1.32)	a
Number of spouses	0 = Yes 5 = No	56.30% 43.70%	2.19 (2.48)	b
Number of children	0 = 5+ children 1 = 4 children 2 = 3 children 3 = 2 children 4 = 1 child 5 = 0 children	10.60% 10.30% 29.70% 29.30% 14.80% 5.30%	2.43 (1.30)	c
Number of grandchildren	0 = 5+ grandchildren 1 = 4 grandchildren 2 = 3 grandchildren 3 = 2 grandchildren 4 = 1 grandchildren 5 = 0 grandchildren	46.20% 15.30% 11.10% 13.50% 7.80% 6.10%	1.40 (1.62)	d
Number of siblings	0 = 5+ siblings 1 = 4 siblings 2 = 3 siblings 3 = 2 siblings 4 = 1 sibling 5 = 0 siblings	19.00% 8.10% 12.60% 19.10% 20.50% 20.60%	2.76 (1.77)	e
Number of friends	0 = 5+ friends 1 = 4 friends 2 = 3 friends 3 = 2 friends 4 = 1 friend 5 = 0 friends	42.40% 6.60% 11.10% 14.50% 8.50% 17.00%	1.91 (1.94)	f
Number of neighbours	0 = 5+ neighbours 1 = 4 neighbours 2 = 3 neighbours 3 = 2 neighbours 4 = 1 neighbour 5 = 0 neighbours	26.80% 7.50% 11.10% 16.80% 14.50% 23.20%	2.54 (1.92)	g
(b) Attendance in presence in the last month	Range	Mean	SD	
Spouse	0 to 5	2.97	2.43	h
Children	0 to 5	3.49	1.54	i
Grandchildren	0 to 5	2.77	1.56	l
Siblings	0 to 5	1.21	1.39	m
Friends	0 to 5	1.46	1.45	n
Neighbours	0 to 5	2.11	1.79	o
(c) Attendance through technology in the last month	Range	Mean	SD	
Spouse	0 to 5	1.36	2.13	p
Children	0 to 5	3.34	1.86	q
Grandchildren	0 to 5	2.61	1.77	r
Siblings	0 to 5	1.86	1.74	s
Friends	0 to 5	1.80	1.68	t
Neighbours	0 to 5	0.96	1.51	u
(d) Community Participation	Range	Mean	SD	
Religious Rites	0 to 5	1.36	1.55	v
Recreational Activities	0 to 5	1.10	1.55	w
Sporting Activities	0 to 5	1.19	1.63	x
Cultural Activities	0 to 5	1.73	1.86	y
Lunches and dinners with relatives	0 to 5	1.57	1.42	z

Note: 5 = never, 4 = once or twice a month, 3 = approximately once a week, 2 = approximately two to three times a week, 1 = almost every day, 0 = every day.

**Table 2:** Attendance of the network of significant relationships before COVID and during the COVID outbreak.

	Before COVID-19	During Outbreak	*Z*	*p*
	Mean (SD)	Mean (SD)		
Sons	3.91 (1.42)	3.49 (1.54)	−14,285 ^a^	0.000
Grandchildren	3.41 (1.45)	2.77 (1.56)	−18,616 ^a^	0.000
Siblings	1.92 (1.66)	1.21 (1.39)	−19,755 ^a^	0.000
Friends	2.40 (1.62)	1.46 (1.45)	−21,261 ^a^	0.000
Neighbours	2.48 (1.88)	2.11 (1.79)	−10,155 ^a^	0.000

Note: ^a^ = Based on positive ranks.

**Table 3:** Attendance of the network of significant relationships during the COVID-19 outbreak in the presence and at a distance.

	In Presence	Via Technological Tools	*Z*	*p*
	Mean (SD)	Mean (SD)		
Sons	3.49 (1.54)	3.34 (1.86)	−1407 ^a^	0.160
Grandchildren	2.77 (1.56)	2.61 (1.77)	−1737 ^a^	0.082
Siblings	1.21 (1.39)	1.86 (1.74)	−13,006 ^b^	0.000
Friends	1.46 (1.45)	1.80 (1.68)	−7391 ^b^	0.000
Neighbours	2.11 (1.79)	0.96 (1.51)	−18,834 ^a^	0.000

Note: ^a^ = Based on positive ranks; ^b^ = Based on negative ranks.

**Table 4:** Means, standard deviations and correlations between the variables included in the study.

	Means (SD)	Range	1	2	3	4	5	6
1. Mental Illness (GHQ)	3.04 (0.50)	1–5	1					
2. Age discrimination in the context of the COVID-19 pandemic (ADCo)	2.75 (0.96)	1–5	0.24 **	1				
3. Loneliness (UCLA)	2.10 (0.47)	1–4	0.46 **	0.24 **	1			
4. Social Isolation (SII)	2.92 (0.64)	0–5	0.25 **	0.03	0.32 **	1		
5. COVID-19 Risk Perception (CoRP)	3.78 (0.83)	1–5	0.26 **	0.36 **	0.16 **	−0.04	1	
6. Fear of COVID-19 (FCV-19S)	3.03 (0.92)	1–5	0.34 **	0.38 **	0.21 **	0.04	0.65 **	1

Note: ** *p* < 0.01.

## Data Availability

The data presented in this study is available from the respective author upon request. The data is not publicly available due to the continuation of the project in this regard.
